# Casein Kinase-1-Alpha Inhibitor (D4476) Sensitizes Microsatellite Instable Colorectal Cancer Cells to 5-Fluorouracil via Authophagy Flux Inhibition

**DOI:** 10.1007/s00005-021-00629-2

**Published:** 2021-09-18

**Authors:** Morvarid Siri, Hamid Behrouj, Sanaz Dastghaib, Mozhdeh Zamani, Wirginia Likus, Sedigheh Rezaie, Jacek Hudecki, Saeed Khazayel, Marek J. Łos, Pooneh Mokarram, Saeid Ghavami

**Affiliations:** 1 Autophagy Research Center, Shiraz University of Medical Sciences, Shiraz, Iran; 2 Department of Biochemistry, School of Medicine, Shiraz University of Medical Sciences, P.O Box: 1167, Shiraz, Iran; 3 Endocrinology and Metabolism Research Center, Shiraz University of Medical Sciences, Shiraz, Iran; 4 Department of Anatomy, School of Health Science in Katowice, Medical University of Silesia, ul. Medyków 18, 40-762 Katowice, Poland; 5 Laryngology Department, School of Medicine in Katowice, Medical University of Silesia, Katowice, Poland; 6 Department of Research and Technology, Kermanshah University of Medical Sciences, Kermanshah, Iran; 7 Department of Pathology, Unii Lubelskiej 1, Pomeranian Medical University, 71-344 Szczecin, Poland; 8 Research Institute of Oncology and Hematology, Cancer Care Manitoba, University of Manitoba, Winnipeg, Canada; 9 Faculty of Medicine, Katowice School of Technology, Katowice, Poland; 10 Department of Human Anatomy and Cell Science, Max Rady College of Medicine, Rady Faculty of Health Sciences, University of Manitoba, Winnipeg, Canada

**Keywords:** 5-Fluorouracil, Casein kinase 1 alpha inhibitor, Colorectal cancer, Combination treatment, Autophagy, Chemoresistance

## Abstract

Adjuvant chemotherapy with 5-fluorouracil (5-FU) does not improve survival of patients suffering from a form of colorectal cancer (CRC) characterized by high level of microsatellite instability (MSI-H). Given the importance of autophagy and multi-drug-resistant (MDR) proteins in chemotherapy resistance, as well as the role of casein kinase 1-alpha (CK1α) in the regulation of autophagy, we tested the combined effect of 5-FU and CK1α inhibitor (D4476) on HCT116 cells as a model of MSI-H colorectal cancer. To achieve this goal, the gene expression of *Beclin1* and MDR genes, *ABCG2* and *ABCC3* were analyzed using quantitative real-time polymerase chain reaction. We used immunoblotting to measure autophagy flux (LC3, p62) and flow cytometry to detect apoptosis. Our findings showed that combination treatment with 5-FU and D4476 inhibited autophagy flux. Moreover, 5-FU and D4476 combination therapy induced G2, S and G1 phase arrests and it depleted mRNA of both cell proliferation-related genes and MDR-related genes (*ABCG2*, *cyclin D1* and *c-myc)*. Hence, our data indicates that targeting of CK1α may increase the sensitivity of HCT116 cells to 5-FU. To our knowledge, this is the first description of sensitization of CRC cells to 5-FU chemotherapy by CK1α inhibitor.

**Graphic abstract:**

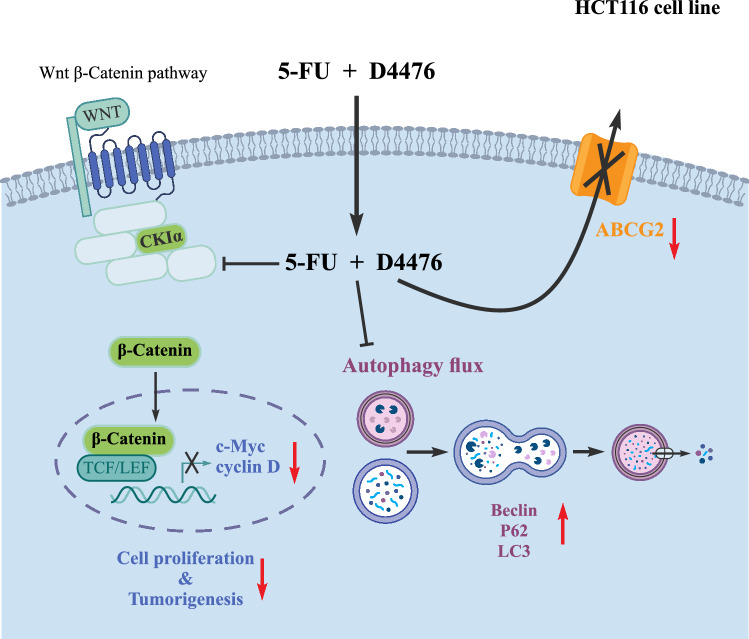

## Introduction

Colorectal cancer (CRC) is among the most common and lethal cancers worldwide (Blondy et al. 2020; Mokarram et al. 2017). CRC treatment is highly dependent on tumor stage. Surgery is commonly used as the primary treatment for CRC, but chemotherapy is also used as an adjunct therapy to reduce the risk of recurrence of stages 1 and 2 cancers after surgery (Reima et al. 2020).

FOLFOX is a neoadjuvant chemotherapy for patients with advanced or metastatic disease including Oxaliplatin, Leucovorin, and Fluorouracil sequential therapy (Symonds and Cohen 2019). FOLFOX is generally more effective as compared to fluorouracil/Leucovorin for patients with stage III colon cancer (Rebuzzi et al. 2020). However, it should be noted that 5-fluorouracil (5-FU) is still the most widely used medication for CRC therapy (Vodenkova et al. 2020). The anticancer effect of 5-FU is mainly mediated through thymidylate synthase inhibition by 5-FU metabolites which consequently interfere with DNA metabolism (Parker and Cheng 1990). Despite the high efficacy of 5-FU-based chemotherapy, an acquired treatment resistance occurs in almost 40% of cases (Longley and Johnston 2005). The adjuvant therapy with oxaliplatin or irinotecan may potentially overcome 5-FU resistance, in some cases (Douillard et al. 2000; Longley et al. 2003). Still, more effective combined therapeutic strategies involving adjuvant medications to ameliorate mechanism involved in resistance to chemotherapy are being sough. Several well-known mechanisms may contribute to drug resistance in cancer, such as apoptosis inhibition (Qie and Diehl 2016), drug inactivation (Mansoori et al. 2017), multi-drug resistance (MDR) via different ATP-binding cassette (ABC) transporters (e.g., ABCB1, ABCG2, ABCB4, ABCC15, ABCC10, and ABCG4) (Fletcher et al. 2016), cell cycle modulators (e.g., c-myc and cyclin D1) (Christowitz et al. 2019), and the autophagy pathway (Desantis et al. 2018; Hombach-Klonisch et al. 2018).

One of the most extensively studied mechanism of cancer drug resistance involves ABC transporters (Khunweeraphong and Kuchler 2021). They facilitate the anticancer agents’ efflux out of cancer cells and decrease the efficacy of treatment in colon and numerous other cancers (Hu et al. 2016). Suppressing ABC transporter protein expression is effective in overcoming 5-FU resistance in CRC, in vitro (Blondy et al. 2020). Studies show that overexpression of ABCG2 is responsible in both innate and acquired MDR phenotypes in cancer stem cells (An and Ongkeko 2009; Hasanabady and Kalalinia 2016).

Besides ABC transporters, the autophagy pathway is another mechanism that contributes to drug resistance in numerous cancers, and plays the key role in the tumor microenvironment (Mele et al. 2020; Shojaei et al. 2020). Activated autophagy results in the lysosomal degradation of damaged organelles and supernary or misfolded proteins, and hence provides energy (Aghaei et al. 2020; Bhardwaj et al. 2017; Klionsky and Emr 2000). The “double-edged sword” action of autophagy in tumor formation and progression is a matter of ongoing research (Ahmadi et al. 2020; Liu and Ryan 2012).

Despite the suppressing role of autophagy in the initial phases of cancer formation, it could also increase cell survival, promote metastasis, and protect cancer cells from environmental- and drug-induced stress (Dastghaib et al. 2020; Samiei et al. 2020). Hence, autophagy may participate in the development of MDR and increase the survival of cancer cells exposed to chemotherapeutic agents (Li et al. 2017). Therefore, autophagy could potentially be a novel target in cancer therapy to overcome drug resistance. Since MDR activation poses a challenge for cancer therapy, hence novel ways to overcome it are an important goals in cancer research (Kwan and Wai 2016).

Two strategies have been reported to inhibit autophagy. One is to inhibit the autophagy at the beginning of the path through Beclin 1 inhibition. The second strategy is to increase the autophagy followed by the accumulation of dysfunctional autophagy vesicles which is called autophagy flux inhibition (Li et al. 2020, 2021). The conversion of LC3I to LC3II and p62 and Beclin 1 proteins are autophagic markers, well-suited for autophagy monitoring (Alizadeh et al. 2018; Emami et al. 2019; Li et al. 2020).

Researchers have found several regulators that stimulate or inhibit autophagy. Recently, casein kinase 1-alpha (CK1α) was discovered as a novel promising autophagy regulator in the lung, breast, multiple myeloma, and colon cancer cells (Cai et al. 2018; Carrino et al. 2019; Cheong et al. 2015; Szyniarowski et al. 2011). The protein kinase CK1 family includes seven isoforms with serine/threonine-specific protein kinase activity (α, β, γ1, γ2, γ3, δ, ε) (Cheong and Virshup 2011). CK1α enzyme regulates diverse signaling processes such as Wnt-signaling, cell cycle, and apoptosis (Lorzadeh et al. 2021; Schittek and Sinnberg 2014). The hallmark of Wnt-signaling cascade is the target gene upregulation; *c-myc*, or *Cyclin D1* are thought to be linked to cell proliferation (Li et al. 2020). Elevated *c-myc* level could induce autophagy; therefore, oncogene targeted therapy in combination with autophagy inhibition may offer novel approach for cancer treatment (Zhang and Cheong 2013). In contrast, the activation of c-myc/miR-150 axis inhibits the autophagic flux and promotes the development of non-small cell lung cancer (Emami et al. 2019). There is no clear evidence on the direct or indirect connection of *c-myc* oncogene and autophagy. Therefore, further research is required to understand the relationship between CK1α, Wnt-signaling, cell proliferation, and autophagy markers.

CK1α may also be critically involved in tumor progression and be overexpressed in CRC (Ren et al. 2020). This would in turn lead to the hypothesis that CK1 isoforms offer interesting targets to develop CK1 isoform inhibitors as novel effective therapeutic approaches to hijack tumors (Amaravadi 2015; Janovská et al. 2020; Knippschild et al. 2014; Liu et al. 2019). A group of small molecules has emerged, such as D4476 that suppresses the CK1α activity (Manni et al. 2017). Recent data shows CK1α inactivation is associated with autophagy inhibition that correlates with the suppression of cancer cell growth, (Carrino et al. 2019) hence, further studies are needed to modulate autophagy via CK1α targeting. Autophagy is an important factor influencing chemotherapy resistance to 5-FU in CRC (Chen et al. 2021; Petroni 2020). Considering that autophagy is also regulated by CK1α (Behrouj et al. 2021), we aimed to examine the possible effect of 5-FU/D4476 co-treatment on HCT116, as a model of 5-FU resistant CRC cells.

## Materials and Methods

### Materials and Reagents

5-FU (50 mg/mL) and RPMI 1640 culture medium were bought from Ebewe Pharma (Belgium) and Invitrogen (Carlsbad, CA, USA), respectively. The D4476, CK1α inhibitor D4476 (ab120220) was obtained from Abcam (Cambridge, MA, USA), and its 10 mM stock solution was kept in DMSO at − 20 °C. Antibodies against human p62 (88588S), LC3B (L7543), and Glycerinaldehyde-3-phosphate-dehydrogenase (GAPDH) (sc-47724) were bought from the cell Signaling Technology (Beverly, MA, USA); Sigma-Aldrich (St. Louis, MO, USA) and Santa Cruz Biotechnology (California, USA), respectively. Anti-rabbit IgG (whole molecule) and anti-mouse IgG (Fab specific) peroxidase conjugated secondary antibodies were provided from Sigma-Aldrich (St. Louis, MO, USA).

### Cell Culture

The HCT-116 cell line was obtained from the cell bank of the Pasteur Institute of Iran. HCT-116 cells were cultivated in the RPMI-1640 medium as previously described (Petitprez et al. 2013).

### MTT Assay

The cytotoxicity of 5-FU and D4476 on HCT-116 cells, was measured by MTT-assay, based on the described protocol (Alizadeh et al. 2017; Ghavami et al. 2011). Briefly, HCT116 cells were plated (2 × 10^3^ cells/well) and covered with ascending concentrations of 5-FU (1.25, 2.5, 5, 10, 20, 40, 80, 100 and 160 μM) and D4476 (1.56, 3.12, 6.25, 12.5, 25 μM) for 24, 48, and 72 h. Then, absorbance was measured at 570 nm, after the cells were kept for 4 h in the presence of MTT reagent (5 mg/ml, 20 μM). The IC50 values were assessed from the cell-survival plots (Aghaei et al. 2020). Combination cytotoxic effect of D4476 with 5-FU was also evaluated using CalcuSyn software (Biosoft).

### Real-Time Polymerase Chain Reaction

Quantitative real-time polymerase chain reaction (RT-PCR) was performed as described previously (Hashemi et al. 2013; Karami et al. 2020). Briefly, the cells were covered with specific concentrations of 5-FU (10 and 1.25 μM), D4476 (5 μM), and the combination of 5-FU and D4476 for 24, 48, and 72 h. The total RNA was isolated by the RNX-Plus RNA extraction kit (Cinnagen, Iran), following the manufacturer’s instruction, and then RNA quantity and quality were assessed by electrophoresis and a Nano Drop spectrophotometer (Thermo Scientific, USA) (260/280 nm ratio). cDNA was made by the cDNA Synthesis Kit (Thermo Fisher Scientific, USA) and amplified in the presence of specific primers (Table 1). The *ABCC3* (GenBank accession no. NM-003786.4), *ABCG2* (GenBank accession no. NM-004827.3), *c-myc* (GenBank accession no. NM-002467.6), *Cyclin D1* (GenBank accession no. NM-053056.3), *Beclin1* (GenBank accession no. NM-003766.4) and *GAPDH* (GenBank accession no. NM-002046.7) primers were obtained from Metabion (Germany). Quantitative RT-PCR was performed in an ABI real-time PCR 7500 system (Applied Biosystems, USA). *GAPDH* was considered as a reference control. The relative quantity of the target genes was calculated, using the delta-delta Ct (2^–ΔΔCT^) method.

**Table 1:** PCR specific primers

Genes	Forward primers	Reverse primers
*ABCC3*	CAACCTCATGTCAGTGGAT	GAAGTAGATCGCCAGGATG
*ABCG2*	TATAGCTCAGATCATTGTCACAGTC	GTTGGTCGTCAGGAAGAAGAG
*c-myc*	CATACATCCTGTCCGTCCAAG	CGCACAAGAGTTCCGTAGC
*Cyclin D1*	CATCCAGTGACAAACCATC	TTATAGTAGCGTATCGTAGGA
*Beclin1*	AGCTGCCGGTTATACTGTTCTG	ACTGCCTCCTGTGTCTTCAATCTT
*GAPDH*	CGACCACTTTGTCAAGCTCA	AGGGGTCTACATGGCAACTG

*ABC* ATP-binding cassette

### Western Blotting

We have performed Western blot analysis of our desired proteins as described in our previous investigations (Ghavami et al. 2009, 2012). To perform Western blot analysis, HCT116 cells were covered with appropriate concentration of 5-FU, D4476, and 5-FU/D4476 for 24, 48, and 72 h. Cells were broken down in NP-40 lysis buffer. The BCA assay kit (Thermo Scientific, USA) was selected to determine the protein concentrations, and then proteins were electrophoresed on SDS-PAGE gel. After blocking with 5% non-fat milk, membranes were covered with primary P62, LC3, and GAPDH (as an internal control) antibodies (1:1000 dilution), in a cold room overnight. Then, the membranes were rinsed with 1X TBST buffer three times for 20 min, and covered with horseradish peroxidase conjugated secondary antibodies (1:2000 dilution) for 2 h at ambient temperature, and then enhanced chemiluminescence reagents (Abcam, USA) were used to develop the blots for 2 min. The chemiluminescent blots were photographed, using the ChemiDoc MP imaging system (Bio-Rad, USA). The densitometer was used for the quantification of the band intensity, using Image lab software, version 5.2.1 (Bio Rad).

### Flow Cytometry

The apoptosis level was examined by flow cytometric analysis with the PI staining protocol. The detail of the procedure has been performed as previously described (Ghavami et al. 2014). In brief, cells were seeded in six well plates and covered with 5-FU (10 and 1.25 μM), D4476 (5 μM), and 5-FU/D4476 for 24, 48, and 72 h. EDTA buffer was used to harvest the cells, and then the cells were spun down at 1500 g for 5 min in a cold centrifuge. Phosphate-buffered saline was used to rinse the cells and then the cells were suspended in a hypotonic PI lysis buffer (1% sodium citrate, 0.1% Triton X-100, 40 μg/ml propidium iodide and 0.5 mg/ml RNase A). Cell nuclei were kept at 37 °C for 30 min and evaluated by flow cytometry.

### Statistical Evaluation

Data are presented as mean ± SD of three independent assays (*n* = 3). One-way or two-way ANOVA was used to determine the statistical differences, followed by Tukey’s or Bonferroni’s post-hoc test. GraphPad Prism 8 software (GraphPad Prism, RRID:SCR_002798) was used for statistical analysis. *P* < 0.05 was considered statistically significant.

## Results

### D4476 Stimulates Cell Death in the HCT-116 Cells

**Fig. 1:**
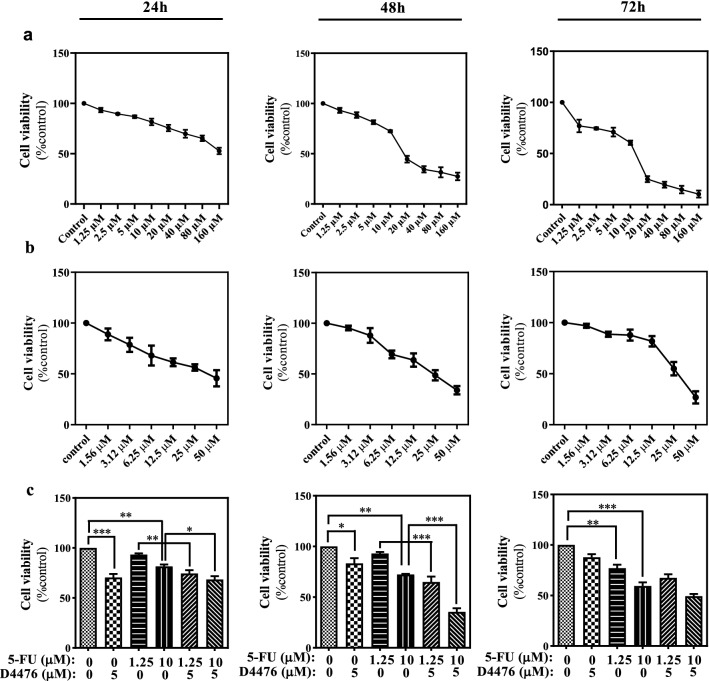
The effect of 5-FU, D4476 and combination of D4476 with 5-FU on the growth of HCT-116 colon cancer cells. The cells were exposed to different concentrations of 5-FU (**a**), D4476 (**b**), and combination thereof (**c**), for 24 h, 48 h and 72 h, and the cell viability, was assessed by MTT assay. Data are reported as mean ± SD of three independent assays (*n* = 3, *P < 0.05; **P < 0.01; ***P < 0.001)

The MTT assay was used to measure the cytotoxic effect of 5-FU and D4476 on HCT-116 cells. As shown in Fig. 1a, 5-FU significantly induced cell death in HCT-116 cells. *P* < 0.001 for concentrations ≥ 5 μM (24 h), *P* < 0.001 for concentrations ≥ 5 μM (48 h), and *P* < 0.001 for concentrations ≥ 1.25 μM (72 h). The treatment of HCT-116 cells with D4476 resulted in significant cell death (Fig. 1b): *P* < 0.001 for concentrations ≥ 6.25 μM (24 h), *P* < 0.001 for concentrations ≥ 6.25 μM (48 h), and *P* < 0.001 for concentrations ≥ 25 μM (72 h). The IC50 values of 5-FU and D4476 at 24, 48, and 72 h are shown in Table 2. Therefore, 1.25 and 10 μM concentrations of 5-FU and 5 μM of D4476 were selected for the combined treatment groups.

**Table 2:** IC50 values of 5-FU and D4476 after 24, 48 and 72 h exposure to cells

Timepoints	IC50 (μM)	
	5-FU exposure	D4476 exposure
24 h	161.6	42
48 h	18.2	24.5
72 h	11.7	30

IC50 values at 24, 48 and 72 h exposure. Data are reported as means ± SD of three independent experiments (*n* = 3)

**Fig. 2:**
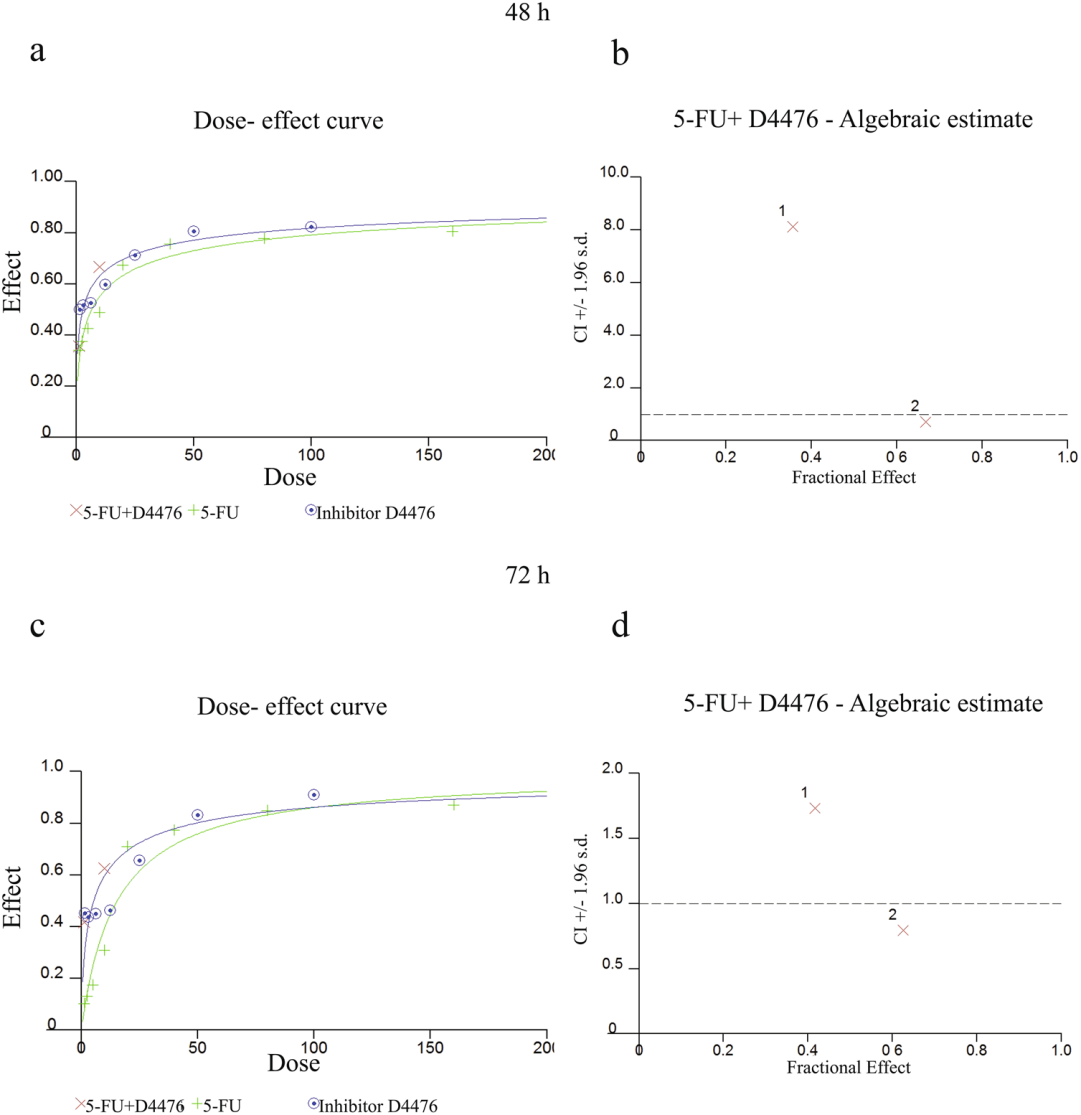
Synergistic interaction of D4476 with 5-FU in HCT-116 colon cancer cells. Dose–effect curve for HCT-116 colon cancer cells (**a**, **c**). CI-fractional effect curves for HCT-116 colon cancer cells (**b**, **d**)

We also measured 5FU/D4476 combination therapy and showed that the combination therapy with 10 µM 5-FU has the most significant effect (Fig. 1c). As shown in Fig. 1c, combination of D4476 with 5-FU (1.25 and 10 µM) induced more cell death compared to time matched 5-FU treatment in HCT-116 cells. The most significant effect was detected in combination of 10 µM 5-FU/5 µM D4476 compared with 10 µM 5-FU alone at 48 h (*P* < 0.001). Synergistic cytotoxic effect of 5FU with D4476 on HCT-116 cell lines was determined by Combination Index (CI) (CI < 1 shows synergistic effect). Our results showed that 5-FU had a synergistic effect in 10 µM concentration at 48 h (Fig. 2a, b) and 72 h (Fig. 2c, d).

### Combination of 5-FU and D4476 Increased the Population of Sub-G1 Phase and S-Phase Arrest

**Fig. 3:**
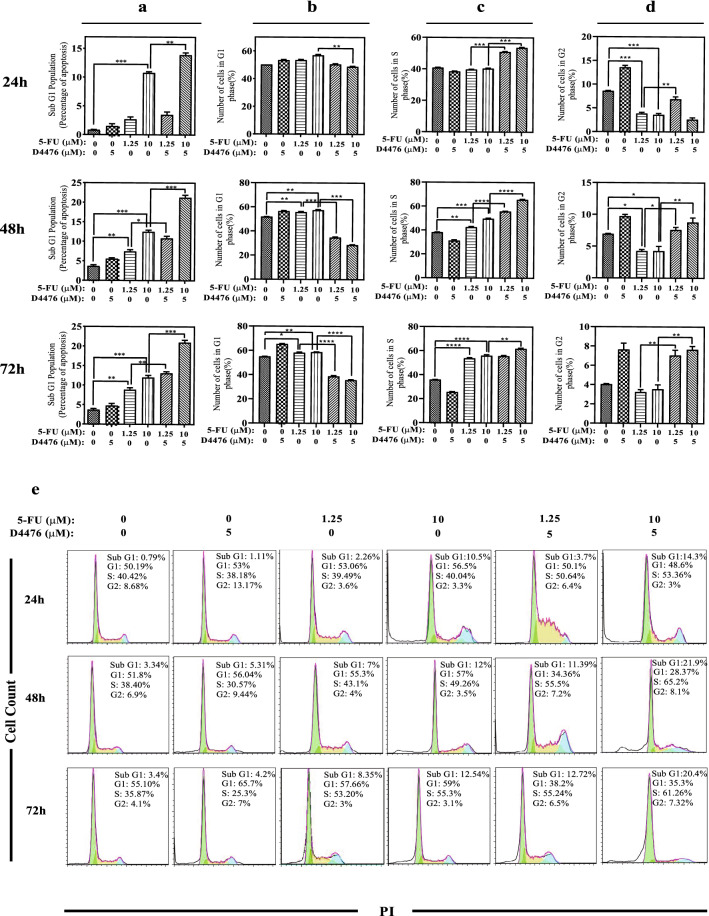
Cell cycle distribution after HCT-116 cells were exposed to 5-FU and D4476 alone or in combination at 24, 48 and 72 h. Quantification of the sub-G1 (**a**), G1 (**b**), S (**c**) and G2 (**d**), population (bars represent percentage of cells [%] in each fraction). Flow cytometry histograms indicating cell cycle distribution and apoptosis (sub-G1) after HCT-116 cells were exposed to 5-FU and D4476 alone or in combination for 24, 48 and 72 h (**e**). Data are reported as means ± SD of three independent assays (*n* = 3, *P < 0.05; **P < 0.01; ***P < 0.001)

Flow cytometry analysis was done to evaluate whether D4476 induces apoptosis in HCT-116 cells. As demonstrated in Fig. 3a and e the percentages of sub-G1 populations were higher in all the combined treatment groups, compared to each of the treatments alone. After 24 h (Fig. 3a), the percentage of sub-G1 populations significantly increased (14.3%) after co-treatment with 10 µM 5-FU and 5 µM D4476 compared with a single treatment of 10 µM 5-FU (10.5%, *P* < 0.01). This increase was not significant for the combination of 1.25 µM 5-FU and 5 µM D4476 (3.7%) compared with 1.25 µM 5-FU (2.26%) alone (*P* > 0.05). After 48 and 72 h (Fig. 3a), combination of D4476 and both concentrations of 5-FU (1.25 and 10 µM) significantly increased the percentages of sub-G1 cell populations (*P* < 0.05 or less) compared with single therapies (11.39% and 12.72% for 1.25 µM 5-FU combined with D4476 and 21.9% and 20.4% for 10 µM 5-FU combined with D4476 after 48 and 72 h, respectively). The result also showed that the combination of 1.25 and 10 µM 5-FU with D4476 increased cell accumulation in S-phase compared with single treatment by 5-FU alone in a time and dose-dependent manner (Fig. 3c and e). After 48 and 72 h, combination of D4476 and both concentrations of 5-FU (1.25 and 10 µM) significantly caused cell accumulation in S-phase compared with 5-FU alone (55.5% and 65.20% for 1.25 µM 5-FU combined with D4476 and 55.24% and 61.26% for 10 µM 5-FU combined with D4476 after 48 and 72 h, respectively; Fig. 3e). As shown in Fig. 3b, combination of both 1.25 and 10 µM 5-FU with D4476 significantly decreased percentage of cells in G1-phase compared with matched control at 48 and 72 h. After 48 and 72 h (Fig. 3d), the percentage of G2 populations significantly increased after co-treatment with 10 µM 5-FU /5 µM D4476 compared with a single treatment of 10 µM 5-FU (*P* < 0.01). This increase was also significant for the combination of 1.25 µM 5-FU/ 5 µM D4476 compared with 1.25 µM 5-FU alone at 24, 48 and 72 h.

### D4476 Augments 5-FU Cytotoxicity Through Autophagy Flux Inhibition

**Fig. 4:**
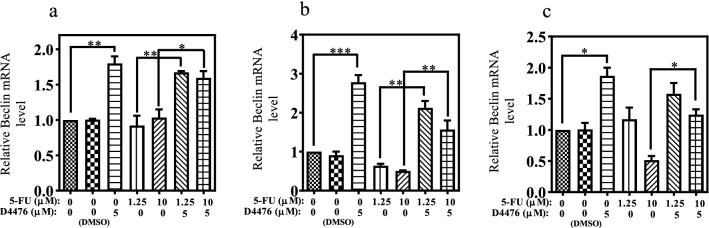
The *Beclin1* expression is upregulated in HCT-116 cells. The relative mRNA expression levels of *Beclin1* in HCT-116 cells for 24 h (**a**), 48 h (**b**) and 72 h (**c**). Data are reported as mean ± SD of three independent experiments (*n* = 3, *P < 0.05; **P < 0.01; ***P < 0.001)

**Fig. 5:**
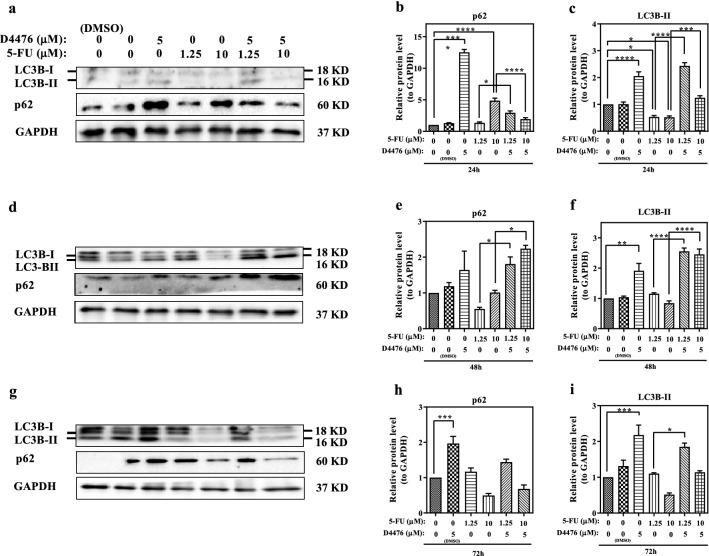
D4476 induced the expression of LC3 and P62 in HCT-116 cells. LC3 and P62 protein levels were analyzed via western blotting for 24 h (**a**–**c**), 48 h (**d**–**f**), 72 h (**g**–**i**). The loading control was GAPDH. Results are reported as mean ± SD of three independent tests (*n* = 3, *P < 0.05; **P < 0.01; ***P < 0.001; ****P < 0.0001)

Since *Beclin1*, LC3, and p62 are routinely used to monitor the autophagy, effects of the combined treatments with 5-FU and D4476 on the expression of *Beclin1*, LC3, and p62 were measured after 24, 48, and 72 h. The *Beclin1* mRNA level was detected by RT-PCR. As shown in Fig. 4, the *Beclin1* mRNA expression level increased in the combined treatment groups compared with 1.25 µM and 10 µM 5-FU alone after 24, 48, and 72 h. The protein expression levels of p62 and LC3β-II were assessed by Western blotting. As shown in Fig. 5, in the combined treatment groups (with both 1.25 and 10 µM 5-FU), LC3β-II expression increased, compared with 1.25 µM or 10 µM 5-FU alone at 24 h (*P* < 0.0001 and *P* < 0.001, respectively), 48 h (*P* < 0.0001) and 72 h (*P* < 0.05 and *P* > 0.05, respectively).

An increased expression of P62 in the cells treated with the 1.25 µM 5-FU and D4476 combination was detected by western blot, compared with the 5-FU alone at 24 h (*P* < 0.05), while the combination of 10 µM 5-FU and D4476 significantly decreased the p62 expression (*P* < 0.0001, Fig. 5a–c). In the combined treatment groups, the p62 expression significantly increased, compared with 5-FU alone at 48 h (Fig. 5d–f, *P* < 0.05). However, this increase was not significant after 72 h.

### Combination of 5-FU and D4476 Downregulates the *ABCG2*, *c-myc* and *cyclin D1* Expression

**Fig. 6:**
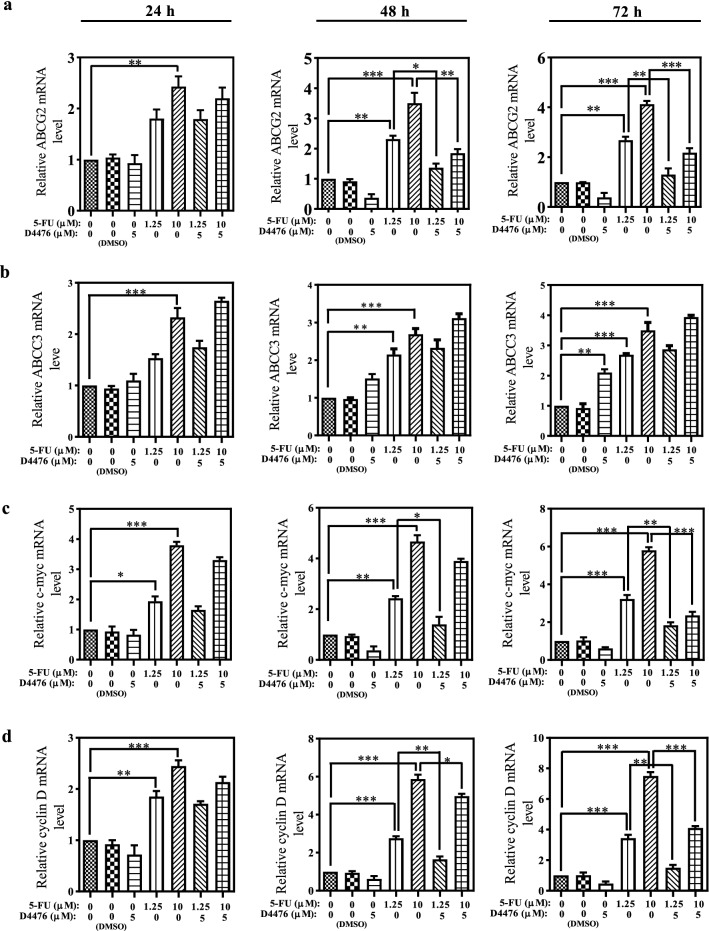
Combination D4476 and 5-FU downregulates the expression levels of ABCG2, c-myc and Cyclin D. The relative mRNA expression levels of ABCG2 (**a**), ABCC3 (**b**), c-myc (**c**) and Cyclin D (**d**) in HCT-116 cells for 24, 48 and 72 h. Data are reported as mean ± SD of three independent assays (*n* = 3, *P < 0.05; **P < 0.01; ***P < 0.001)

To investigate the impact of 5-FU combined with D4476 on the expression of ABC transporters, the mRNA levels of *ABCC3* and *ABCG2* transporters were tested by RT-PCR (Fig. 6). As shown in Fig. 6a, 1.25 µM 5-FU alone had no significant effect on the *ABCG2* expression at 24 h. However, it significantly increased the *ABCG2* expression at 48 h (2.31-fold, *P* < 0.01) and 72 h (2.68-fold, *P* < 0.01), compared with the control groups. Upon 10 µM 5-FU treatments, the *ABCG2* mRNA expression was upregulated after 24 h (2.43-fold, *P* < 0.01), 48 h (3.5-fold, *P* < 0.001) and 72 h (4.12-fold, *P* < 0.001). Compared with treatment with 1.25 µM or 10 µM 5-FU alone, co-treatment of D4467 with 5-FU significantly reduced *ABCG2* expression after 48 h (*P* < 0.05 for 1.25 µM 5-FU, and *P* < 0.01 for 10 µM 5-FU) and 72 h (*P* < 0.01 for 1.25 µM 5-FU and *P* < 0.001 for10 µM 5-FU).

Compared to the control groups, 1.25 µM 5-FU alone significantly increased the *ABCC3* expression after 48 h (2.14-fold, *P* < 0.01) and 72 h (2.69-fold, *P* < 0.001), but had no significant effect after 24 h. In 10 µM 5-FU treatments, the *ABCC3* mRNA expression was upregulated at 24 h (2.32-fold, *P* < 0.001), 48 h (2.68-fold, *P* < 0.001) and 72 h (3.49-fold, *P* < 0.001). The 5-FU combined with D4476 also induced a slight increase in the *ABCC3* expression (*P* > 0.05) (Fig. 6b). To determine the Wnt signaling target genes, we also assessed the *c-myc* and *cyclin D1* that control cellular transcription and cell cycle regulation in colon cancer cells.

RT-PCR showed that 5-FU alone stimulated the *c-myc* and *cyclin D1* expression in a time-dependent manner (Fig. 6c, d). The 1.25 µM or 10 µM 5-FU alone significantly increased the *c-myc* expression after 24 h (1.93-fold, *P* < 0.05 for 1.25 µM 5-FU and 3.79-fold, *P* < 0.001 for 10 µM 5-FU), 48 h (2.43-fold, *P* < 0.01 for 1.25 µM 5-FU and 4.67-fold, *P* < 0.001 for 10 µM 5-FU) and 72 h (3.22-fold, *P* < 0.001, for 1.25 µM 5-FU and 5.8-fold, *P* < 0.001, for 10 µM 5-FU).

As shown in Fig. 6c, the combination of D4476 with 1. 25 µM 5-FU significantly reduced *c-myc* expression at 48 h (*P* < 0.05) and 72 h (*P* < 0.01), compared with 5-FU alone. Significant downregulation was observed for the *c-myc* expression in the cells treated with 10 µM 5-FU and D4476 combination after 72 h (*P* < 0.01).

1.25 µM and 10 µM 5-FU alone significantly increased the *cyclin D1* expression after 24 h (1.85-fold, *P* < 0.01 for 1.25 µM 5-FU and 2.44-fold, *P* < 0.001 for 10 µM 5-FU), 48 h (2.76-fold, *P* < 0.001 for 1.25 µM 5-FU and 5.87-fold, *P* < 0.001, for 10 µM 5-FU) and 72 h (3.44-fold, *P* < 0.001 for 1.25 µM 5-FU and 7.51-fold, *P* < 0.001 for 10 µM 5-FU) (Fig. 6d).

As shown in Fig. 6d, the combination of D4476 with 1.25 µM 5-FU significantly reduced *cyclin D1* expression after 48 h (*P* < 0.01) and 72 h (*P* < 0.01), compared with 5-FU alone. Significant downregulation was observed for the *c-myc* expression in the cells treated with 10 µM 5-FU and D4476 combination after 48 h (*P* < 0.05) and 72 h (*P* < 0.001).

## Discussion

In this study, combined 5-FU/D4476 decreased *ABCG2, cyclin D1* and *c-myc* gene expression and also increased the levels of autophagy markers including LC3-II, p62 and Beclin1 compared with the 5-FU alone in HCT116 human CRC cells. To our knowledge, this is the first description of sensitization of colorectal cancer cells to 5-FU chemotherapy by CK1α inhibitor.

Chemotherapy is the commonly used for treating colon cancer, leading to the extension of survival rates (Ghavami et al. 2014). 5-FU is one of the most frequently used anticancer agents, and the first choice in the treatment of colon cancer, but drug resistance limits its efficacy (Jung et al. 2020; Longley et al. 2003; Tai et al. 2013). Accordingly, it is clinically important to develop new chemotherapeutic approaches counteracting drug resistance. The autophagy pathway is a known mechanism contributing to drug resistance in cancer (Levine and Kroemer 2008; Mele et al. 2020). Autophagy could be induced by many external and internal stimulators (e.g., chemotherapy, oxidative stress, starvation, etc.) to maintain cellular homeostasis (Brun et al. 2020; Katayama et al. 2007; Klionsky and Emr 2000). Nevertheless, over-activation of autophagy may lead to a specific kind of cell death, called programmed cell death type II or autophagy cell death (Maiuri et al. 2007). In chemotherapy, autophagy can either increase cell survival (chemotherapy-modulated autophagy) or promote cell death in some cases (Li et al. 2009; Shojaei et al. 2020).

**Fig. 7:**
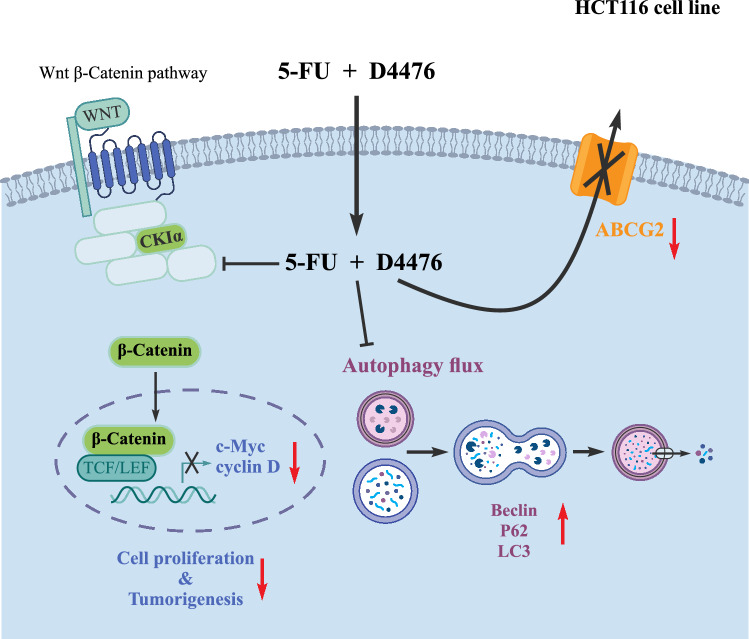
Schematic representation of main multi-target effects of combination D4476 and 5-FU at the sub-cellular level. The combination of both drugs affects the Wnt/β-catenin pathway through the inhibition of CKIα, by autophagic flux upregulation (when compared to 5-FU alone), and by ABCG2 transporter downregulation

Our results indicated that both autophagy markers (LC3β-II and *Beclin1*) were significantly diminished in human CRC cells (HCT116) after treatment with 5-FU alone. These results are consistent with another study (Yao et al. 2017) showing that SNUC5/5-FUR, as 5-FU resistant SNUC5 colon cancer cells, have lower levels of autophagy, compared with their parental cells. Moreover, Yang et al. (2018) suggested that chemotherapy-induced autophagy improved the tumor-killing effect and proliferative inhibition (Fig. 7). Hence, the attenuation of autophagy in response to 5-FU therapy may contribute to the survival for cancer cells, and to evade cell death (Kroemer and Jäättelä 2005; Maiuri et al. 2007).

It has been recently described that CK1α plays a role in upregulating the autophagy pathway in several cancer cell lines (Behrouj et al. 2021; Cai et al. 2018; Carrino et al. 2019; Cheong et al. 2015; Szyniarowski et al. 2011). Furthermore, it has been suggested that CK1α overexpression, may serve as a negative prognostic marker for CRC. Hence it could serve as a new promising therapeutic target (Richter et al. 2018). To improve this therapeutic strategy, and mitigate possible side effects one would likely need to develop and test several isoform-specific inhibitors of CK1.

We have found that D4476 augmented the inhibitory effect of 5-FU on the viability of HCT116 cells. Moreover, D4476 treatment enhanced 5-FU-induced apoptosis of HCT116 cells. Also, 5-FU/D4476 increased *Beclin1*, LC3 II, and p62 levels. When LC3-II levels increase with p62 accumulation, autophagy is impaired (Wang et al. 2018). Therefore, 5-FU/D4476 increased the autophagy flow followed by the accumulation of dysfunctional autophagy vesicles. Bcl-2 is able to bind to *Beclin1* and consequently prevent pre-autophagosome structure assembly and inhibit autophagy (Marquez and Xu 2012). Increased *Beclin1* level may indicate that Bcl-2 may become predictably ineffective, and therefore, it can be concluded that autophagy plays an impact role in cell death independent from apoptosis. However, the lack of information about Caspases 3, 9 and Bcl-2 alterations, is a limitation in this study that should be investigated in future studies to predict the exact mechanism of cell death. Moreover, 5-FU/D4476 increased in *Beclin1* and a decrease in p62 during 24 h, indicating that autophagy had started at 24 h without any inhibition of autophagy flux because of p62 consumption. Interestingly, the inhibitory effect of D4476 increased after 48 and 72 h, while more autophagosome is accumulated. In this regard, it was found that CK1α inhibition led to the overexpression of autophagy genes in RAS-mutated colon cancer cell lines (Cheong et al. 2015) through stabilization of transcription factor FOXO3a (regulator of autophagic flux). Recently, CK1α has been recognized as a negative regulator of oncogenic RAS-induced autophagy, further strengthening our argument that CK1α-regulated autophagy can be a promising therapeutic target (Xu et al. 2020).

Carrino et al. (2019), suggested that CK1α inhibition with the CK1α inhibitor D4476 resulted in impaired degradation of autophagosomes in multiple myeloma, which was likely related to its effect on the acidification of the lysosomes. D4476 also led to the accumulation of autophagic markers LC3B-II and p62 in MM cell lines and induced cell death as a consequence of an accumulation of ineffective autophagic vesicles. Furthermore, studies have shown that wogonin and 3-methyladenine inhibit sorafenib-induced autophagy and improve sorafenib sensitivity in human hepatocellular carcinoma cells (Kiruthiga et al. 2020; Rong et al. 2017). Tubeimoside-I could also enhance the efficacy of chemotherapeutic agents to treat CRC cells and overcome drug resistance by disrupting the autophagy flux (Yan et al. 2019). All in all, these results are in support of our observation that D4476 could enhance chemotherapeutic efficacy of 5-FU by inhibiting autophagy flux.

We also noted that mRNA expression levels of *ABCG2*, *cyclin D1*, and *c-myc* significantly decreased in D4476 and the combined treatment groups, whereas *ABCC3* expression increased. Some studies have shown that the overexpression of ABC transporters’ genes, c*yclinD1* and *c-myc,* results in the development of MDR (Biliran et al. 2005; Kugimiya et al. 2015; Robey et al. 2007). Hence, it seems that downregulation of *ABCG2*, *cyclin D1*, and *c-myc* could improve sensitivity to 5-FU. Studies show that increased resistance because of higher *ABCG2* expression has been associated with increased autophagic flux. In this study, *ABCG2* knockdown reduced autophagy activity in resistant cells, confirming that enhanced autophagy is dependent on *ABCG2* (Ding et al. 2016). It was found that reduced *c-myc* expression is involved in resistance to 5-FU via the downregulation of ABCB5 (Kugimiya et al. 2015). It was also suggested that the expression level of *ABCG2* and *ABCB5* decreased following *c-myc* silencing and sensitized colon cancer stem cells to chemotherapy (Zhang et al. 2019). According to Kobayashi et al. (2016), inhibition of the Wnt signaling pathway leads to the increase *ABCC3* mRNA levels which in turn reduces the sensitivity to 5-FU in HCT-116 cells but has no significant effect on 5-FU sensitivity in HT29 and SW620 cell lines. Considering the discrepancy of ABCC3 behavior in different cell lines, further study is required to clarify if ABCC3 inhibition might be a promising targeted therapy.

β-catenin is the main transcription factor regulating *cyclinD1*, *c-myc* and the gene expression of ABC transporters (Kim et al. 2017). Aberrant activation of Wnt/β-catenin pathway and increased β-catenin stability are associated with the overexpression of *cyclinD1*, *c-myc,* and ABC transporters (e.g., *ABCG2*, *ABCC1*) (He et al. 1998; Tetsu and McCormick 1999). CK1α increases β-catenin stability through phosphorylation at Ser268 and Ser269 (Peters et al. 1999; Vinyoles et al. 2014). Moreover, the inhibition of CK1α enhances the drug cytotoxicity in multiple myeloma by reducing the β-catenin level (Manni et al. 2017). Therefore, it seems that a reduction in the β-catenin level might be a reason for the reduction in the mRNA levels of *cyclinD1*, *c-myc*, and *ABCG2*, in response to CK1α inhibition.

Our results indicate that 5-FU/D4476 increased in the percentage of cells in sub G1 (apoptotic) phase. mRNA level of *cyclinD1* was detected via real time analysis and *cyclin D1* reduction could also partly be explained via sub-G1 induction. In addition, 5-FU/D4476 synergistically inhibited cell viability, which reduced the clinically required dose of 5-FU as well as drug resistance. In line with this study, the inhibition of CK1α by siRNA or D4476 treatment triggered cell cycle arrest and an increase in the percentage of cells in sub-G1 (apoptotic) phase in human colorectal polyps and multiple myeloma cells (Jiang et al. 2018; Manni et al. 2017). In this regard, Dimethylfumarate also has anti-tumorigenic properties on colon cancer cells and sensitizes cells to radiation through apoptosis, which is accompanied by cell cycle arrest in the G0/G1 phase and reduces cyclin D1 expression (Kaluzki et al. 2019). In addition, combination of 5-FU/D4476 increased the cytotoxic effect of 5-FU by repressed cyclin D1 level and enhanced S-phase arrest. D4476 also induced G2/M arrest and 5-FU/D4476 synergistically enhanced G2 arrest. In this regard, Guoyi Wu and colleagues found that UCN-01, a antitumor agent, inhibits proliferation in the hepatoma cells by inducing S and G2/M phase arrest, but not G1/S arrest (Wu et al. 2013). Cell’s progression from G1- to S-phase is controlled by Cyclin D1, but its suppression during S phase is needed to allow synthesis of DNA. High level of cyclin D1 prevents incorporation of 5-FU into the DNA and the early S-phase cell cycle is blocked. In this regard, Larasati and colleagues found that NF-κB suppresses DNA synthesis by increasing cyclin D1 levels. It seems that cyclin D1 inhibits 5-FU action by inhibiting DNA synthesis in the early S phase. In line with the current study, they showed that PGV-1 suppressed cyclin D1 expression by inhibiting NF-κB and increased S-phase block with 5-FU. The combination of PGV-1 with 5-FU enhances the cytotoxic effect of 5-FU by increasing 5-FU S-phase arrest and suppressing cyclin D1 levels. PGV-1 also enhanced G2/M arrest and inhibited NF-κB transcriptional activity (Meiyanto et al. 2018).

Although, the results of cellular viability by MTT and PI assay have almost the same pattern, but there are also slight differences, in which case the flow cytometry results are more accurate, pointing at limitations of MTT assay. Because of such limitations, it seems that for analysis of cell cytotoxicity, cell viability, cell proliferation and apoptosis, we need to perform a series of tests, including MTT assay, Trypan blue counting, flow cytometry as well as RT-PCR, or better: Western blot detection of pro-apoptotic proteins level.

Beside direct cytotoxic activity of 5-FU towards (colorectal) cancer cells, it exhibits yet another indirect effect through modulation of immune system. It sensitizes cancer cells towards immune effector CD8 T cells (Ghiringhelli and Apetoh 2015). It also decreases the number of myeloid-derived suppressor cells (MDSC) through the activation of apoptosis characterized by the activation of the key effector caspases-3 and -7 (Vincent et al. 2010). MDSC exhibit low activity of thimidine kinase, hence, they are highly sensitive towards 5-FU.

Eradication of colorectal cancer stem cells is critical for a successful therapy (Jahanafrooz et al. 2020; Jaromy and Miller 2021; Wang et al. 2020). While this paper does not directly focus on the issue, since active autophagy is important for the maintenance of stem cells in general, including cancer stem cells (Auger et al. 2021) D4476 may potentially affect colorectal cancer stem cells maintenance. This relevant issue will be resolved upon completion of our relevant project.

In conclusion, targeting CK1α might be a robust approach to increase sensitivity of colorectal cancer cells to 5-FU through Wnt signaling pathway, G2 and S phase arrests as well as G1 arrest, depletion of the mRNA levels of *ABCG2*, and autophagy flux inhibition. In addition, 5-FU/D4476 treatment may expedite the effectiveness of the treatment protocols in colon cancer.

## Data Availability

Data are available upon request.

## Abbreviations

5-FU: 5-Fluorouracil
ABC: ATP-binding cassette
CI: Combination Index
CK1α: Casein kinase 1-alpha
CRC: Colorectal cancer
GAPDH: Glycerinaldehyde-3-phosphate-dehydrogenase
MDR: Multi-drug resistant
